# Enhancing water quality and well-being of *Penaeus vannamei *(Boone, 1931) in Inland saline systems using mixed biochar amendments

**DOI:** 10.1038/s41598-024-84973-1

**Published:** 2025-01-08

**Authors:** Newmei Tachangliu, Vidya S. Bharti, Chalungal T. Amal, Tao Kara, Saurav Kumar, Pankaj Kumar, A. K. Verma, Arun Konduri, Swaraj Adakney, Shamika Sawant

**Affiliations:** ICAR-Central Institute of Fisheries Education, Mumbai, 400061 India

**Keywords:** Biochar, Sediment, Water Quality, Growth, *Penaeus vannamei*, Inland saline water, Zoology, Environmental sciences

## Abstract

An experiment was conducted for 60 days in a 500L capacity FRP tank containing inland ground saline water (fortified to a level of 50% potassium) with one control (sediment) and three treatments; T1(Paddy Straw Biochar (PSB) in sediment), T2 (Banana Peduncle Biochar (BPB) in sediment), and T3 (PSB + BPB in sediment). Biochar (100 g) was amended with sediment (25 kg) at 9 tons/ha. Shrimps of average weight 5 ± 0.03 g were stocked at 60 juveniles/m^3^ and were fed at satiation levels using commercially available feed. Compared to control, in biochar contained treatments the ammonia levels were reduced, the pH, alkalinity, calcium-magnesium ratio, and potassium in water, were increased significantly. Growth metrics reveal a significant increase in final body weight, weight gain percentage, PER, HPSI, SGR, and reduced FCR (within 1–1.26) in biochar-treated groups with the highest survivability (92%) was observed in T3, which is mixed biochar amended sediment. At the end of the experiment, shrimp organs (hepatopancreas, gills, and muscles) and serum were sampled for tissue enzymes and serum profiles, respectively. The mean levels of lipase, amylase and protease varied significantly, and in biochar treated groups significant reduction in the activities of oxidative stress enzymes (SOD and CAT in Hepatopancreas) were recorded in comparison to control. For the serum, higher hemocyanin (0.33 ± 0.01mMolL^-1^) was observed in mixed biochar amended treatment (T3) and the least in control. In addition, the glucose level in the serum was significantly dropped in biochar-amended groups indicating reduced stress levels, while it increased in control. T3 was found to be the best, among all, in improving growth performance and maintaining the water quality. Even though biochar-amended treatments demonstrated positive outcomes regarding inland saline water quality, growth metrics, and well-being of the *P. vannamei* compared to control, a deeper analysis is necessary to understand the underlying mechanisms determining these beneficial effects of biochar.

## Introduction

Indian fisheries have testified that a paradigm change from capture-dominated fisheries towards culture-based fisheries has facilitated the development of a sustainable blue economy. The marine exports from India are dominated by shrimp, which account for 73% of the overall export value, with 90% contributed by cultured shrimp^1^. Among different shrimp species, the whiteleg shrimp (*Penaeus vannamei)* has recently gained prominence due to its success in aquaculture. Nevertheless, the lack of credible land and water is an emerging issue with the further expansion of these aquaculture systems.

The world’s food and nutritional security are gravely threatened by groundwater salinization. On about 1 billion hectares of land worldwide, salinization is acknowledged as the greatest threat to environmental and economic health. Salt affects 6.74 million hectares in India, of which 12 lakh ha are in northern India’s non-coastal Indo-Gangetic plains. Given that most agricultural plants are vulnerable to salinity caused by high salt concentrations in the soil, and that the amount of land affected by it is growing daily, salinity is a critical environmental factor limiting crop productivity^2^. Considering this, the current focus is to sustainably cultivate crops and other food items on degraded or otherwise utilizable land.

Recently, aquaculture has emerged as a promising alternative for utilizing inland saline lands and water resources. Whiteleg shrimp (*P. vannamei*) farming in low-saline aquaculture systems depends upon ions present in the culture medium. Pond seepage is the main issue in inland saline aquaculture in addition to a deficiency of K^+^ and variable Ca:Mg ratio. Therefore, it is imperative to balance the ionic composition in the inland ground saline water by supplementing with exogenous substances as needed or system need to be stimulated to release ions trapped in the sediment into the water column. Recent studies reported the utilization of biochar as a soil supplement to increase nutrient status in saline soils, particularly in terms of improving the availability of minerals like calcium, magnesium, and potassium^3^.

The amount of Agro-waste generated on global scale is about 1 billion tons yearly^4^. There is a potential for exploring these unwanted resources for the biochar production using appropriate technology. According to Zhang et al.^5^, Biochar is a porous material, with characteristic high carbon content and a range of functional groups. It presents a feasible option for handling solid waste and can be produced through pyrolysis of diverse biomass residues like agricultural waste^6^. Applying biochar to soil influences soil texture, particle size distribution, bulk density, and porosity. In addition to rich mineral content, biochar has unique properties like large surface area and the presence of surface oxygen functional groups^7^ which makes it an excellent adsorbent material relevant to aquaculture applications^8,9^. The banana peduncle has a substantial amount of cellulose, lignin, and hemicellulose, making it a good candidate for biochar production. Banana peduncle biochar has 42.55% potassium, which is the highest potassium content reported among various biochar types^10^. Also, the use of ash-rich rice husk biochar can be beneficial in boosting soil organic carbon, cation exchange capacity, and accessible potassium, lowering soil bulk density^11^. The ease of availability of paddy residue and higher potassium in banana peduncle were the factors exploited while mixing these biochar types. Since the waste feedstock does not compete with energy and food crops or arable land, producing biochar from agricultural post-harvest wastes is a viable waste management strategy, and is the best fit to circular economy concept.

In inland saline shrimp farms, Fertilizer-grade muriate of potash (KCl) and magnesium chloride are utilized for the ionic manipulation of raw IGSW with potassium (K^+^) and magnesium (Mg^2+^), respectively, aiming to enhance shrimp survival and growth^12^. It appears that the use of chemical fertilizers not only be costly but also causes adverse impacts on the environment^13^; thus, adding ions such as Mg^2+^ and K^+^ to the feed may provide a more cost-effective solution^12^. Alternatively, biochar can be used as a sediment supplement which can increase the potassium level in inland saline water in addition to improving water quality parameters, and there by the productivity of the culture system can be enhanced^8^. Thus, converting banana peduncles and paddy straw into consistent, long-lasting biochar can be an eco-friendly and cost-effective solution in managing the crop residues along with exploring its beneficial properties such as a mineral supplement and as a potential adsorbent.

There are only a few reports on using biochar in agricultural and degraded soils to improve soil characteristics. Likewise studies on the application of biochar and its advantages in aquaculture and related fields are still scarce. So, the focus of this study is to integrate these breaches in literature in order to assess the potential of mixed biochar as a suitable and efficient technology to enhance water quality parameters, growth performance, and overall health of *P. vannamei*.

## Materials and methods

### Preparation of biochar

The raw Paddy Straw (PS) was obtained from the farmers at Palghar district, Maharashtra, India . Left over after the harvest was collected directly from the field. Similarly the left over Banana Peduncle (BP) was obtained from local banana chips making units located at Mumbai, and air-dried for one week till the moisture content was less than 12%. The biochar was made in a 2.5-kg internal biomass holding capacity electrical heating kiln. At 400 °C, the kiln was operated for 1 h and 30 min. The recovery percentage of biochar was calculated as:

Biochar Yield (%) = $$\frac{Amount of Biochar (g) \times 100}{Amount of dried biomass residue (g)}$$

### Characterisation of biochar

The pH and EC values of biochar were measured by mixing distilled water with biochar samples (1 g of biochar: 20 mL of deionized water) and agitating at low speed for 1.5 hours^14^. For total element nutrient analysis, 0.5 g each of dried biomass (BP and SB) and biochar sample was digested in a mixture of nitric (HNO_3_) and perchloric acids (3:1 ratio)^15^. The moisture and ash content of the biochar was measured using the gravimetric method. The carbon and nitrogen content of the biochar was estimated using a CHNS elemental analyzer and functional groups were identified with Fourier Transform Infrared (FTIR) spectroscopy (SHIMADZU, FTIR 4100). Further the Energy-dispersive X-ray spectroscopy (EDS) technique was used to determine the percentage composition of elements in biochar. 

### ***Preparation of potassium (K***^+^***) fortified Inland Ground Saline Water (IGSW)***

The fortified inland ground saline water (F-IGSW) of salinity 10 ppt, with K^+^ levels 50% that of seawater can be made by supplementing raw inland ground saline water (R-IGSW) with muriate of potash (KCl) according to the formula given by Davis et al.^16^.

### Procurement of seeds and conditioning

The specific pathogen-free (SPF) *P. vannamei* PL10 were airlifted from Sree Sai Hatcheries, Yarrayapeta, Andhra Pradesh, India, to the experimental site, Rohtak, Haryana, India. To acquire juveniles 5 ± 0.03 g for the experiment, post-larvae (PL10) were conditioned in inland saline ponds enriched with potassium levels 100% comparable to seawater at a salinity of 15 ppt. According to the feeding chart, the PLs were fed with commercial starter feed crumbles (GROWEL Feeds Pvt. Ltd., Andhra Pradesh, India).

### Setting of tanks and preparation of control and treatments

A completely randomized design was followed for the experiment in wet lab under controlled conditions with one control and 3 treatments in triplicate in 500L capacity FRP tanks. All the tanks were disinfected using potassium permanganate, washed with water, and left for air drying. The tanks were labeled to know the water level and the treatment given. 25 kg of dry inland soil was added to each tank. Biochar was amended to the sediment by proper mixing, followed by making the soil-biochar slurry. In each tank, 100 g of biochar was used (biochar application rate at 9-ton ha^-1^). In treatments, T1; consisted of sediment bed was amended with paddy straw biochar, T2; sediment was amended with banana peduncle biochar, and T3; the mixed biochar (paddy straw biochar + banana peduncle biochar in 1:1 ratio) amended sediment. In control, no such amendment was done. After 5 days, all the tanks were filled with IGSW fortified to a level of 50% with muriate of potash (potassium levels 50% equivalent to that of seawater). To each tank, the experimental animals (shrimp) were released at a stocking density of 60 shrimps/m^3^ (30 juveniles per tank). Shrimps of each experimental group were fed to satiation level four times a day. In each tank, topping of water was done using F-IGSW (having potassium levels 50% equivalent to that of seawater) during the halfway period of the experiment. Throughout the experiment ambient temperature was 28–36℃.

### Water quality parameters

The APHA method^17^ was used to examine the water quality parameters.

### Sampling and data collection for growth metrics and physiological parameters of Penaeus vannamei

The entire biomass of shrimps was measured to determine their starting weight. The number of surviving shrimps in each replicate was noted at the conclusion of the 60-day experiment, and the final body weight (FBW) of shrimps were assessed after 24 h of fasting. The following parameters were determined using the standard formula given by Jana et al.^18^.

$$\text{WG }(\text{\%})=\frac{\text{final wet weight }\left(\text{g}\right)-\text{Initial wet weight }\left(\text{g}\right)}{\text{Initial wet weight }(\text{g})}\times 100$$

$$\text{SGR}\left(\text{\%}\right)/\text{day}=\frac{\text{Ln }(\text{final weight})-\text{Ln }(\text{initial weight})}{\text{Experimental period }(\text{days}) }\times 100$$

$$\text{FCR}=\frac{\text{Feed intake }(\text{dry weight in g})}{\text{Body weight gain }(\text{wet weight in g})}$$

$$\text{PER}=\frac{\text{Body weight gain }(\text{wet weight in g})}{\text{Protein intake }(\text{dry weight in g})}$$

$$\text{Survival }\left(\text{\%}\right)=\frac{\text{Total number of alive shrimps}}{\text{Total number of stocked shrimps}}\times 100$$

$$\text{HPSI }\left(\text{\%}\right)=\frac{\text{Wet weight of the hepatopancreas }(\text{g})}{\text{Wet weight of the shrimp }(\text{g})}\times 100$$

### Sample preparation for enzyme assay

From all the experimental tanks, 5 shrimps were collected and anesthetized with clove oil (50 µlL^-1^). Shrimp were then dissected and tissues viz., hepatopancreas, gills, and muscle were taken. Tissue homogenate was prepared in PBS using Teflon coated mechanical homogenizer. The entire procedure was done in ice-cold conditions. Homogenized samples were centrifuged at 8000 rpm for 10–15 min at 4 °C. The supernatant was collected in vials and kept in a deep freezer for the enzyme assay.

### Serum collection

Random sampling was done to collect shrimps from each tank, and a 1 mL syringe (No. 26) was inserted to the ventral sinus of the shrimp to withdraw hemolymph which was then transferred into 2 mL microcentrifuge tubes without anticoagulant. The collected hemolymph was allowed to clot for 1 h and centrifuge at 5000 rpm for 10 min. The supernatant (light blue) was collected with a micropipette and transferred to another microcentrifuge tube. The collected serum samples were stored at -20 °C until analysis. Further, the serum sample was used for the estimation of biochemical parameters.

### Hemocyanin measurement

A spectrophotometer (Hach Ltd., Japan) was used to detect the absorbance at 335 nm after 100 µL of hemolymph were instantly diluted with 900 µL of distilled water in a 10 mm quartz cuvette. With an extinction value of E = 17.26 established by reference to the 74,000 Da functional component, the concentration of hemocyanin was computed and expressed as mmol L^-1^^19^.

### Quantification of tissue protein

Estimation of protein in the different tissue homogenates was carried out by the Bradford method^20^.

### Digestive enzymes activity

The casein digestion technique, as reported by Drapeau^21^, was used to quantify the protease activity in the tissue homogenate. Amylase activity was calculated using the di-nitro- salicylic acid (DNS) method. The titrimetric technique of Cherry and Crandell^22^ was used to evaluate the lipase activity.

### Metabolic enzymes activity

The technique established by Wooten^23^ was used to estimate AST and ALT activity in hepatopancreas and muscle tissue homogenates.

### Stress enzyme activity

The oxidation of the epinephrine-adrenochrome transition by the enzyme served as the basis for the estimation of superoxide dismutase activity (SOD) in gill and hepatopancreas tissue homogenates^24^. The method reported by Takahara^25^ was used to measure the catalase activity (CAT) in hepatopancreas tissue homogenates.

### Statistical analysis

Statistical package SPSS version 25.0 for windows was used to statistically evaluate the data. The means of the various experimental groups in the experiment were compared using one—way ANOVA and Duncan’s multiple range tests and a significant difference was found at the 5% level of probability (p < 0.05). The data were represented as Mean ± standard error (SE).

## Results

### Characterisation of biochar

#### Elemental composition of biochar

**Fig. 1:**
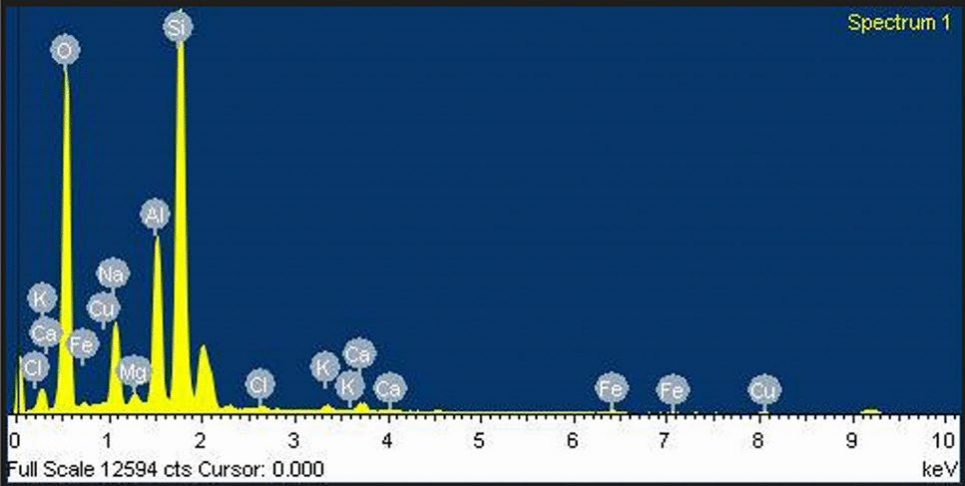
Energy-dispersive X-ray spectroscopy (EDS) of Paddy Straw Biochar (PSB).

**Fig. 2:**
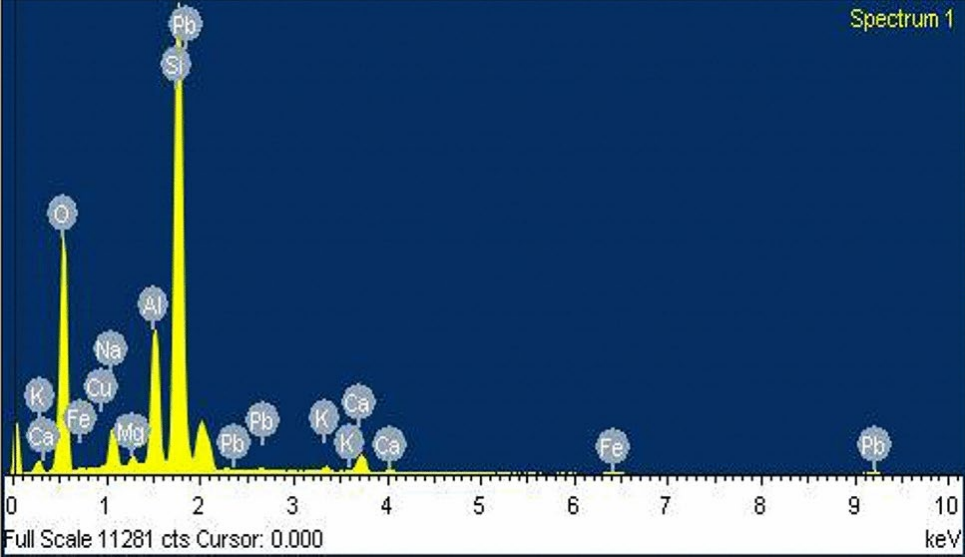
Energy-dispersive X-ray spectroscopy (EDS) of Banana Peduncle Biochar (BPB).

**Fig. 3:**
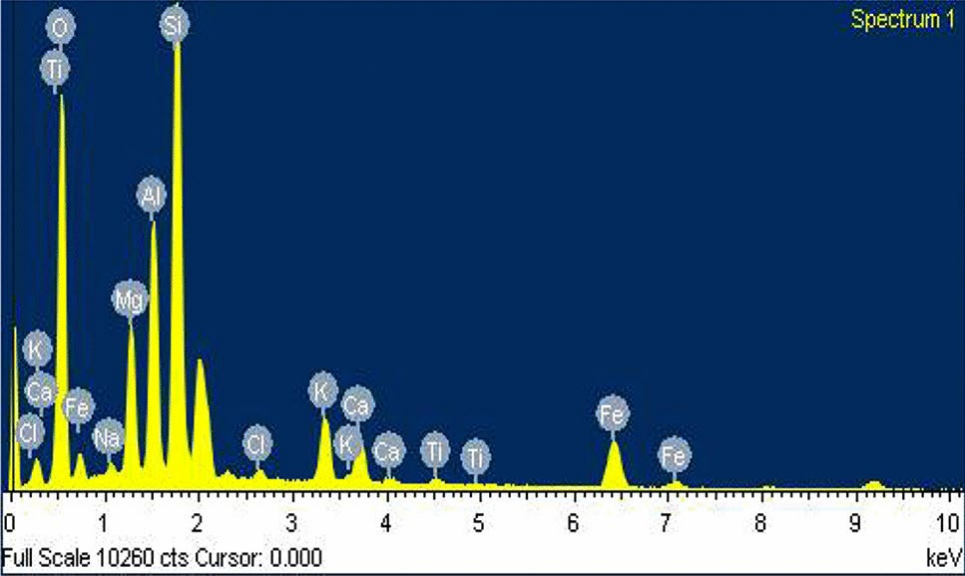
Energy-dispersive X-ray spectroscopy (EDS) of mixed biochar (PSB + BPB).

The elemental composition of biochar was determined by adopting the EDS technique (Figs. 1, 2, 3). The Table 1 presents the percentage composition of each element. In all the biochar samples, Oxygen was found to be significantly high, and the relative abundance of few minor elements ranged from high to low in sequence Si > Al > K > Ca. Although this trend was true in mixed biochar (PSB + BPB), the PSB had weight percentage of minerals in the order Si > Al > Ca > K and for the BPB; Si > Al > K > Ca. The analysis shows that mixed biochar has has relatively higher potassium content of 4.91% followed by banana peduncle biochar (2.89%) and sugarcane bagasse biochar (0.73%).

**Table 1:** SEM–EDS of different biochars used in the study.

Element	Weight percentage (%) in PSB	Weight Percentage (%) in BPB	Weight Percentage (%) in Mixed Biochar
O K	49.94	44.76	39.77
Na K	6.53	3.52	0.55
Mg K	0.60	0.59	5.89
Al K	9.92	8.73	9.30
Si K	29.10	36.81	20.96
Cl K	0.33	0.79	0.67
K K	0.73	2.89	4.91
Ca K	1.21	1.67	3.21
Fe K	1.29	0.25	0.77

PSB- Paddy straw biochar, BPB- Banana peduncle biochar, Mixed biochar- PSB + BPB.

Paddy straw biochar (PSB) and banana peduncle biochar (PSB) were characterized to determine their physicochemical properties. The results are summarized in Table 2.

**Table 2:** Characteristics of biochar pyrolyzed at 400ºC

Physicochemical properties	PSB	BPB
WHC (%)	135	300
BD (g/cm^3^)	0.296	0.75
Biochar recovery (%)	21	46
pH	8.7	8.9
EC (dS m^-1^)	5.9- 6.33	7.3
Ash content (%)	38	18
Carbon (%)	13.06	27.1684
Nitrogen (%)	0.64	0.8689

PSB- Paddy straw biochar, BPB- Banana peduncle biochar.

### Fourier transfer infrared spectroscopy analysis of sediment

The FTIR peaks with their corresponding functional groups in control and biochar-treated sediment after 60 days of the experimental period are shown in Table 3. Sediment contains both non-polymeric hydroxyl (-OH^NP^) and aromatic hydroxyl (-OH^A^) functional groups. The methyl (-CH_2_) and carbonate (-CO_3_^2-^) groups were present in all the sediments, and aromatic hydroxyl (− OH^A^) was exclusive to banana peduncle biochar and mixed biochar which are not present in control and paddy straw biochar amended sediment.

**Table 3:** FTIR peaks and functional groups in the final sediment.

Wave number range (cm^-1^)	Functional groups	C	T1	T2	T3
3670–3610	− OH^A^			3620.58	3621.04
				(− OH^A^)	(− OH^A^)
3600–3220	− OH^NP^	3358.54 (− OH^NP^)	3397.31 (− OH^NP^)		
2935–2915	CH_3_ S–S	2922.54 (-CH_3_) S–S	2922.54 (-CH_3_) S–S	2923.42 (-CH_3_) S–S	
2175–2140	-SCN	2169.77	2169.78	2158.90	2169.77
		(-SCN)	(-SCN)	(-SCN)	(-SCN)
1690–1590	-C = N-	1671.23	1668.84		
		(-C = N-)	(-C = N-)		
1490–1410	-CH_2_	1428.52	1438.13	1436.14	1434.57
	-CO_3_^2 -^	(-CH_2_)	(-CH_2_)	(-CH_2_)	(-CH_2_)
		(-CO_3_^2-^)	(-CO_3_^2-^)	(-CO_3_^2-^)	(-CO_3_^2-^)
1100–1000	PO_4_^2-^	1021.48	1023.68	1019.88	1021.05
		(PO_4_^2-^)	(PO_4_^2-^)	(PO_4_^2-^)	(PO_4_^2-^)
1100–900	= C-H	1021.48	1023.68	1019.88	1021.05
	-SiO_4_^4-^	(= C-H)	(= C-H)	(= C-H)	(= C-H)
		(-SiO_4_^4-^)	(-SiO_4_^4-^)	(-SiO_4_^4-^)	(-SiO_4_^4-^)
600–500	-C-I	531.27 (-C-I)			
500–430	S–S	471.45	469.52	470.52	470.22
		(S–S)	(S–S)	(S–S)	(S–S)

A-Aromatic, NP-Non polymeric. Control: no biochar; T1, indicates Paddy Straw Biochar (PSB) application in sediment; T2, Banana Peduncle Biochar (BPB) application in sediment and T3, sediment amended with mixed biochar (paddy straw biochar + banana peduncle biochar).

The stretching vibrations of hydroxyl (OH) groups at 3800–3300 cm^-1^ are recognized as the crucial region of the spectrum for the identification of aluminous minerals. Illite absorption peaks at 3630 cm^-1^ and has a little potassium in its structure^26^. The treatments T2 and T3 have peaks at 3621 cm^-1^ which indicates the presence of potassium in it, which was not available in control and T1

### Physicochemical parameters of the water

**Fig. 4:**
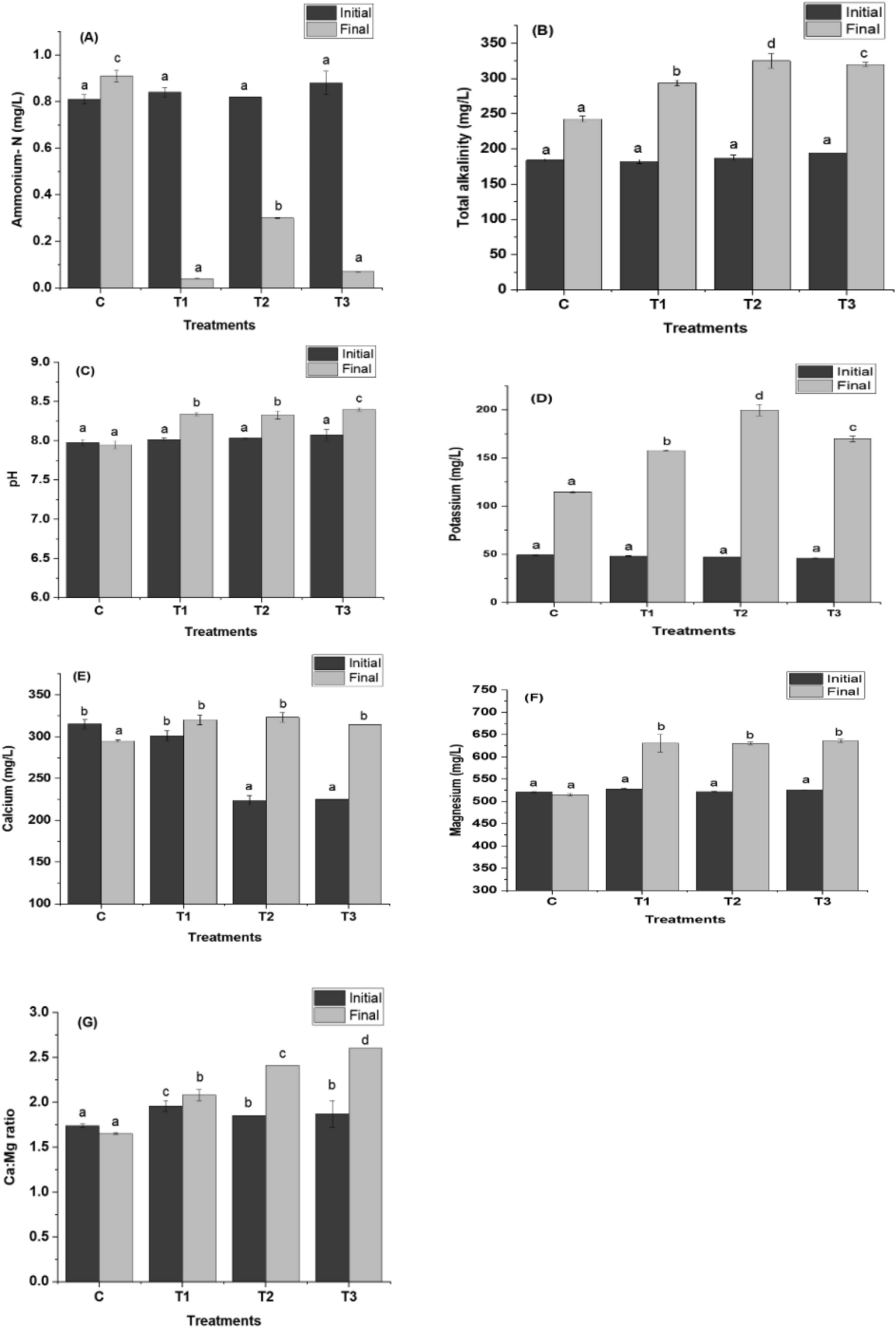
Physicochemical parameters of the water (**A**) Ammonium-N (mg L^-1^), (**B**) Total Alkalinity (mg L^-1^), (**C**) pH, (**D**) Potassium (mg L^-1^), (**E**) Calcium (mg L^-1^ ), (**F**) Magnesium (mg L^-1^), (**G**) Ca:Mg ratio. Control: no biochar; T1, indicates Paddy Straw Biochar (PSB) application in sediment; T2, Banana Peduncle Biochar (BPB) application in sediment and T3, sediment amended with mixed biochar (paddy straw biochar + banana peduncle biochar).

The physicochemical parameters of inland saline water treatments, i.e. ammonium, pH, total alkalinity, potassium, magnesium, and calcium were analyzed at 15-day interval, and ammonia levels were measured every 7 days interval of the experiment. There was a decrease in ammonium-N (mg L^-1^) value in all the treatments from 1^st^ day to the 60^th^ day except for the control. At the end of the experiment, it was found that the ammonium-N in biochar treatments (T1, T2, and T3) were apparently reduced, also T1 which is a paddy straw biochar shows the maximum reduction to 0.04 units (Fig. 4), contrary to the control, where the concentration of ammonia was increased.

There was an observed increase in pH values across all the biochar treatment groups during 60 days of experiment. The highest values of pH (8.41 ± 0.02^c^) were recorded in T3, and the lowest value (7.95 ± 0.05^a^) in control. There was a significant increase in total alkalinity in control and treatments from the initial value. The alkalinity at the end of the experiment ranges from 325 ± 2.88^d^ ppm (in biochar amended treatment group T2) to 242.67 ± 0.04^a^ ppm (in control). The potassium (mg L^-1^) content of water in different treatments increased significantly; 229.16% in T1, 323.78% in T2, and 267.96% in T3 from the initial day of observation till the end of the experiment (Fig. 4), with the highest levels of 200 ± 0.6^d^ mg L^-1^ in T2_,_ and lowest (115 ± 0.8^a^ mg L^-1^) in control.

An elevation of magnesium ions concentration was seen as progression of the experiment. The magnesium level (mg L^-1^) of the water in biochar amended treatments increased significantly on the final day, but as for the control, the level of magnesium was reduced. There were considerable changes in calcium levels between control and treatments. The biochar amended sediment group (T1, T2, T3) exhibited notably higher levels of calcium as compared to control, whereas the control shows no increase in calcium level. In addition, compared to the control, biochar- amended treatment groups exhibited a higher calcium-magnesium ratio (1.00:2.60 in T3).

### Growth metrics of Penaeus vannamei

The various growth metrics of *Penaeus vannamei* in different treatments on 60^th^ day were summarised in Table 4. The final body weight (ranges from 15.92 ± 0.36 g to 19.23 ± 0.05 g) and weight gain percentage of *P. vannamei* in the treatments varied from highest to lowest in the order T3 > T2 > T1 compared to the control. At the end of the experiment, the highest increase in WG % (252.62 ± 6.64%) was observed in T3 and the lowest (131.3 ± 6.3%) in control. The SGR ranges from 1.20 ± 0.05% day^-1^ to 1.85 ± 0.03% day^-1^ with the highest SGR of 1.85% day^-1^ seen in T3 whereas the lowest of 1.20% day^-1^ was recorded in control respectively.

**Table 4.** Growth parameters of *Penaeus vannamei.*

Parameters	C	T1	T2	T3
Average body weight Initial (ABWI) (g)	5.78 ± 0.00 ^a^	5.78 ± 0.01^a^	5.79 ± 0.00^a^	5.76 ± 0.00^a^
Average body weight Final (ABWF) (g)	15.92 ± 0.36^a^	17.94 ± 0.10^b^	18.61 ± 0.65^b^	19.23 ± 0.05^b^
Survival Percentage (%)	80 ± 2.88^a^	83.33 ± 1.66^b^	86.66 ± 1.66^b^	91.66 ± 1.66^c^
Specific Growth Rate (SGR) (% day^-1^)	1.20 ± 0.05^a^	1.57 ± 0.03^b^	1.69 ± 0.02^c^	1.85 ± 0.03^d^
Feed Conversion Ratio (FCR)	1.90 ± 0.09^c^	1.26 ± 0.04^b^	1.15 ± 0.02^a^	1.01 ± 0.05^a^
Protein Efficiency Ratio (PER)	1.5 ± 0.04^a^	1.7 ± 0.07^b^	1.8 ± 0.03^b^	1.9 ± 0.04^c^
Hepatopancreatic Somatic Index (HPSI) (%)	2.79 ± 0.07^a^	3.14 ± 0.06^b^	3.35 ± 0.03^c^	4.02 ± 0.03^d^

Control: no biochar; T1, indicates Paddy Straw Biochar (PSB) application in sediment; T2, Banana Peduncle Biochar (BPB) application in sediment and T3, sediment amended with mixed biochar (paddy straw biochar + banana peduncle biochar). Mean values (Mean ± S.E.) in each row not sharing a common superscript are significantly different (p < 0.05).

The survival percentage of *P. vannamei* differed significantly among the treatments and control. The highest survival percentage (91.66  ± 1.66^c^) was observed in mixed biochar treatment, and the lowest (80  ± 2.88^a^ %) was observed in control. Similarly, the hepatopancreatic somatic index (HPSI) values was highest in T3 (4.02  ± 0.03^d^ %), while the lowest value of 2.79  ± 0.07^a^ % observed in control. The feed conversion ratio (FCR) of *P. vannamei* in biochar treatment shows a significant (p < 0.05) reduction but elevation in PER accounted on the final day of observation (day 60) are shown in Table 4. The FCR ranges from 1.01  ± 0.05^a^ to 1.9  ± 0.09^c^ with the highest FCR (1.90) seen in control and the lowest (1.01) in T3 (mixed biochar treatment). As for the PER, highest value (1.90  ± 0.04^c^) was recorded in biochar amended treatment groups T3, meanwhile control having the least value of 1.50  ± 0.04^a^.

### Digestive enzymes activity

**Fig. 5:**
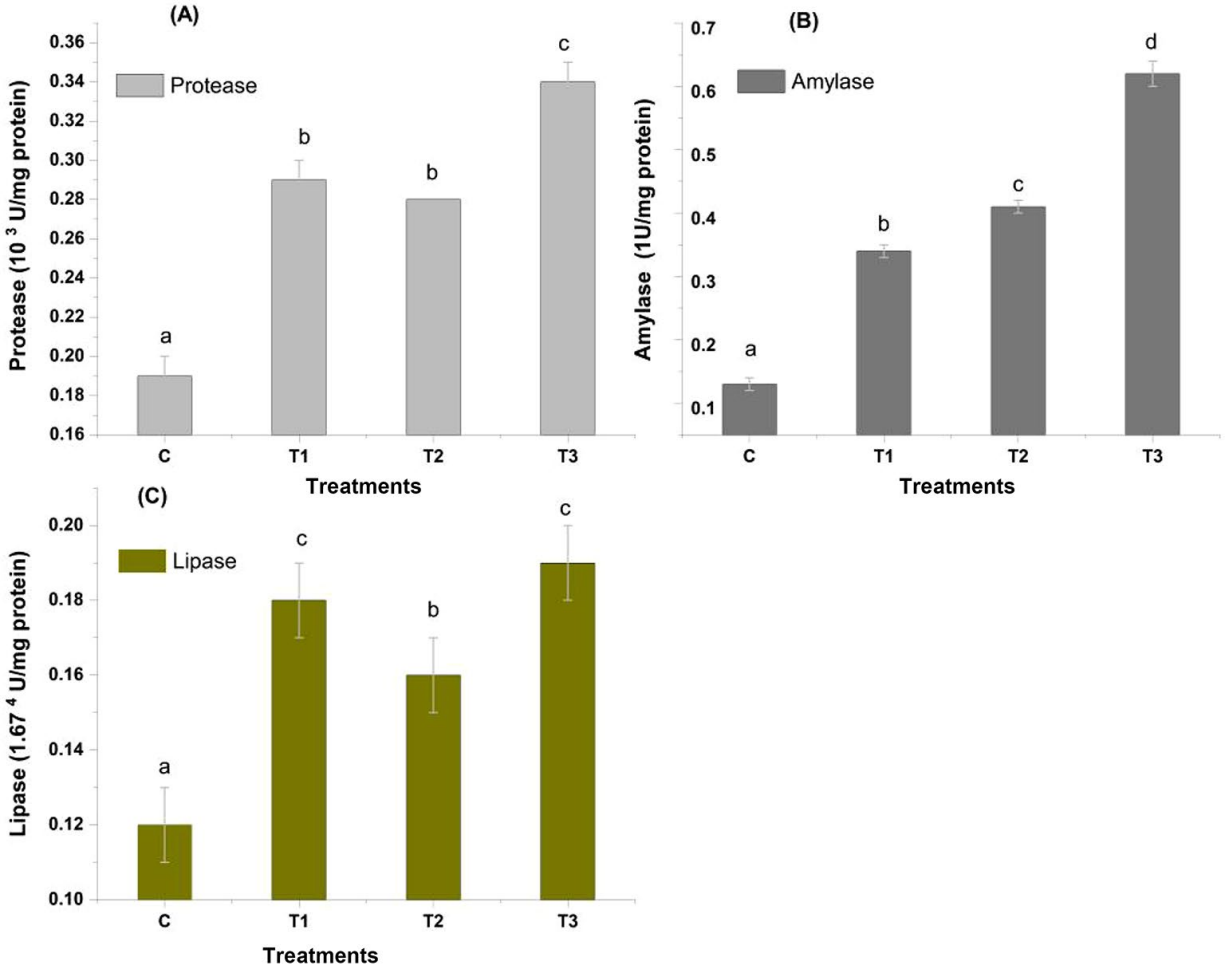
Digestive enzymes activity in *P. vannamei* **(A)** Protease (10^3^ U/mg protein), **(B)** Amylase (1U/mg protein), **(C)** Lipase (1.67^4^ U/mg protein). Control: no biochar; T1, indicates Paddy Straw Biochar (PSB) application in sediment; T2, Banana Peduncle Biochar (BPB) application in sediment and T3, sediment amended with mixed biochar (paddy straw biochar + banana peduncle biochar).

The hepatopancreatic protease activity varied significantly (p < 0.05) among the experimental groups (Fig. 5). Protease activity in the mixed biochar amended group (T3) observed to be the highest (0.34 ± 0.01^c^ mol tyrosine released min^-1^ mg^-1^protein), the least (0.19 ± 0.01^a^ mol tyrosine released min^-1^ mg^-1^ protein) was observed in control. The amylase levels were found to be highest in biochar amended group T3 (0.62  ± 0.02^d^ mol maltose released min^-1^ mg^-1^ protein), with the least observed in control respectively (Fig. 5). As for the lipase activities the mean values were observed significantly (p < 0.05) higher (0.19  ± 0.01^c^) in the mixed biochar applied treatment, followed by T1, T2, and control (0.18   ± 0.01^c^, 0.16  ± 0.01^b^, and 0.12  ± 0.01^a^ U min^-1^ mg^-1^ protein) respectively (Fig. 5).

### Protein metabolic enzymes activity and Oxidative enzymes activity

**Fig. 6:**
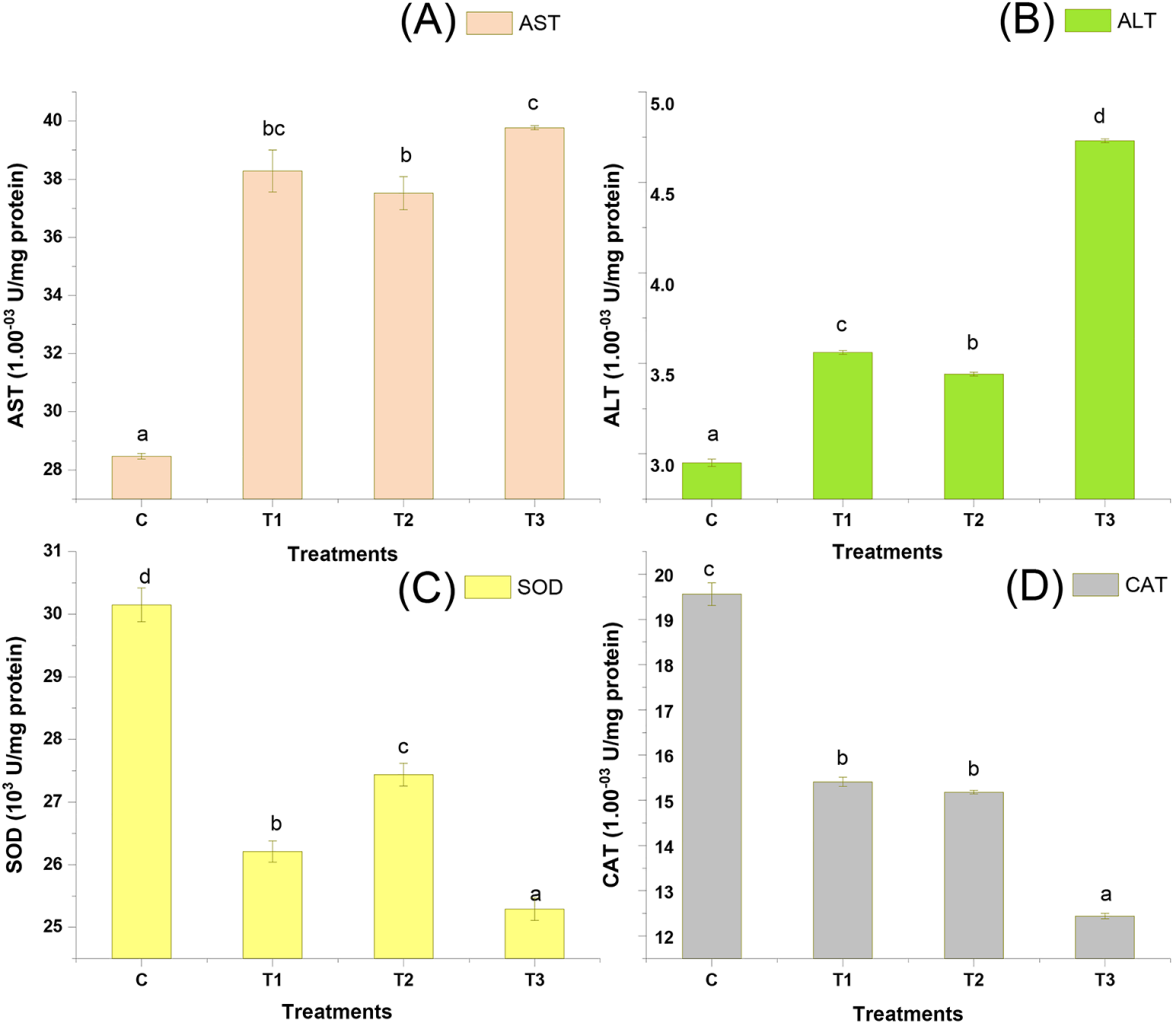
Protein metabolic enzymes activity and Oxidative stress enzymes activity in *P. vannamei* **(A)** AST (Hepatopancreas) (1.00^–03^ U/mg protein), **(B)** ALT (1.00^–03^ U/mg protein), **(C**) SOD (10^3^ U/mg protein), **(D)** Catlase (1.00^–03^ U/mg protein). Control: no biochar; T1, indicates Paddy Straw Biochar (PSB) application in sediment; T2, Banana Peduncle Biochar (BPB) application in sediment and T3, sediment amended with mixed biochar (paddy straw biochar + banana peduncle biochar).

The hepatopancreatic AST and ALT activity (Fig. 6) was observed to be significantly (p < 0.05) higher in mixed biochar treatment (T3), followed by T1 and T2 which was also biochar treatments, and the least was observed in control. The Superoxide dismutase (SOD) activity and Catalase (CAT) activity in the hepatopancreas of *P. vanname*i juveniles was found to be substantially increased (p < 0.05) in control as compared to the treatments (Fig. 6).

### Haemato-biochemical parameters

**Fig. 7:**
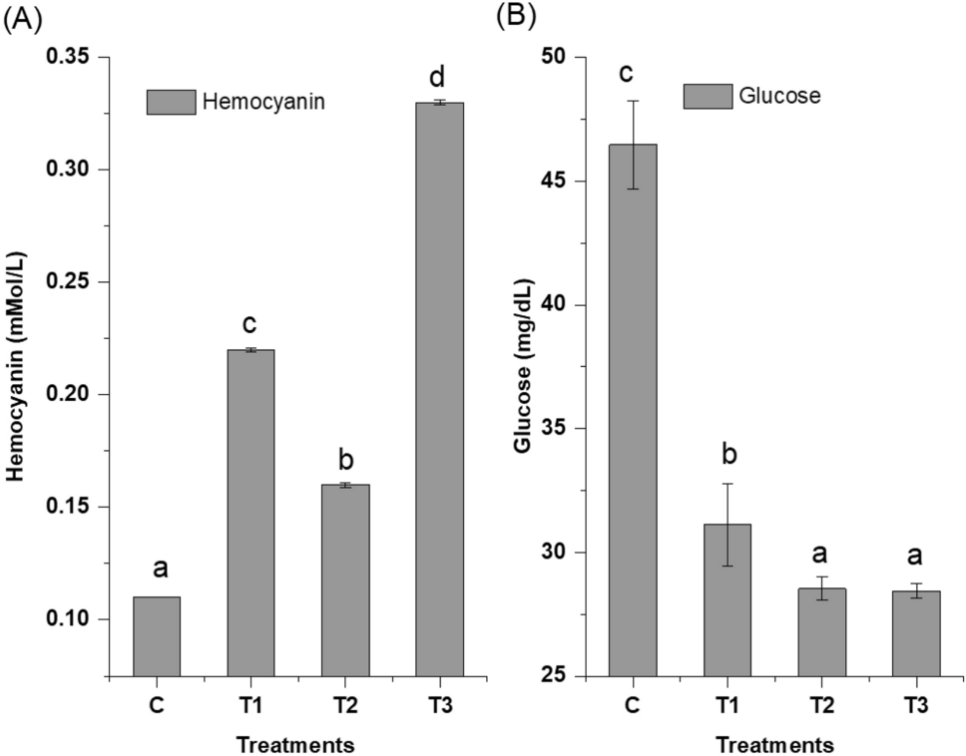
Hemolymph parameters of *P. vannamei* (**A**) Hemocyanin (mMol/L), (**B**) Glucose (mg/dl). Control: no biochar; T1, indicates Paddy Straw Biochar (PSB) application in sediment; T2, Banana Peduncle Biochar (BPB) application in sediment and T3, sediment amended with mixed biochar (paddy straw biochar + banana peduncle biochar).

In control and treatments, hemocyanin concentration differed significantly (Fig. 7). The highest concentration of hemocyanin (0.33 ± 0.01^d^ mMol L^-1^) was observed in mixed biochar treatment, however in control, the concentration was only 0.11 ± 0.01^a^ mMol L^-1^. In control a significantly higher (p < 0.05) serum glucose level was observed (having a value of 46.46 ± 1.77^c^ mg dl^-1^), however among all the treatments, in mixed biochar treatment the serum glucose level was lowest (28.44 ± 0.30^a^ mg dl^-1^) (Fig. 7).

## Discussion

The Paddy Straw and Banana Peduncle biochar was characterized to know their physiochemical properties. The yield of PSB and BPB were 21% and 46% respectively when the pyrolysis conditions were, 400℃ at a residence time of 90 min. The variation in the recovery rate of biochar depends on raw material, pyrolysis conditions (temperature, and time)^27^. It is known that yield decrease with increase in pyrolysis temperature, yield is found to be higher at lower temperature^28^. Consistent to the findings by Kamara et al.^29^ and Abdullah et al.^30^, the physicochemical characteristics of the prepared biochar shows an alkaline pH and higher EC. Regarding the mineral contents, BPB had a higher potassium level ^31^ compared to PSB (Table 1). This was further verified by EDS analysis and FTIR (Table 1, Table 3). The mineral composition of biochar may also depend on raw material as well as locations of raw material from where they are procured.

Due to environmental factors impacting fishes physical condition, growth performance, and yield, the management of water quality conditions is important, especially if the fish are to be produced in aquaculture systems^32^. Biochar is assessed as an effective means for ammonia removal from various systems^33^. In control, the concentration of ammonia increased towards the end of the experimental period, however the biochar treatments demonstrated decreasing levels. This is a positive accomplishment, given that there was only topping of water was done during the trial to make up the evaporative loss. Additionally, it was noted that the water in the culture tanks with biochar-added sediment did not have an odor. Atkinson et al.^34^ found enhanced NH_4_^+^ binding after biochar addition. Surface oxygen groups are the cause of NH_4_^+^ adsorption, according to Spokas et al.^7^. The FTIR spectra also reveal the presence of surface oxygen groups in PSB and BPB (Table 3). It might be the reason for the reduced ammonia content in treatments T1, T2, and T3, compared to control. The increase of ammonium-N in the control water column may be caused by heterotrophic bacteria decomposing soil organic matter including uneaten food and faecal matter.

The capacity of water to buffer or counteract variations in acidity is known as alkalinity. The USEPA classifies pH, which has a recommended range of 6.5 to 8.5, as a secondary pollutant. A high alkaline sodium hydroxide (NaOH) and an unstable (H_2_CO_3_) carbonic acid are created when the dry saline sand interacts with water, which results in the hydrolysis of sodium carbonate (Na_2_CO_3_) resulting in fluctuation in alkalinity and pH^35^. The PSB biochar has more ash content^36^, and banana peduncle biochar is alkaline ^37^ which could be the reason for the rising alkalinity level in the water column in biochar amended tanks. In experimental tanks, water pH significantly increased due to increased primary hydroxide ions from sodium hydroxide and ammonia-N. Also, the alkaline nature of biochar increased pH in biochar-amended sediment treatments. The biochar is known for OH function groups, the FTIR analysis also confirms the presence of OH functional groups in biochar and sediment.

The essential, shortfall mineral potassium in ISW was improved by adding biochar to the sediment ^8,38^. However, Potassium ion availability also depends on soil properties and types of biochar and its raw material, from where it is produced. The potassium level increased highest in T2 (113.70%), and the lowest in control (62.99%) at the end of the experiment, respectively (Fig. 4). The magnesium levels in all the treatments varied significantly with the lowest value was observed in control, while T1 had a higher magnesium level, which is PSB treatment. Similarly, biochar treatments had highest calcium levels while control had the least. The recommended calcium-magnesium value is 1:3 for better survival, growth rate and production of *Penaeus vannamei*^39^. During the experimental period, in all the treatments and control, the value was found to be in the normal range. But a better Ca-Mg balance was observed in biochar applied treatment groups. The ion concentration in water is often influenced by the abundance and availability of sediment cation and anion. Biochar abundant in available ions alters the pH system and influences the confined ions and percolates into the system^40^.

Growth results from dietary nutrient accumulation in the bodies of animals, including finfish and shellfish as well as the surrounding medium. The growth matric reveals that, among the biochar treatments there were significant improvements in the growth performance of *P. vannamei* measured in terms of increased final body weight (g), WG%, SGR and PER, and decreased FCR. Water quality factors such as pH, potassium, Ca: Mg ratio, and ammonia all are essentially chemical factors in shrimp aquaculture determining the growth, survival, and biochemical composition of the shrimp ^8,41^. Complex interrelationships between these parameters can have significant implications on pond productivity, water quality, and shrimp health, to fluctuate over time. Throughout the experiment the water quality in biochar treatments were conducive for shrimp culture. The study also highlights the importance of potassium and calcium – magnesium ratio in water for growth and survival of shrimp. In addition, Najmudeen et al.^42^ related an improved water quality to the enhanced growth of Tilapia when biochar was applied to the sediment.

The physiological and metabolic condition of shrimp, especially concerning liver health and the activity of digestive enzymes involved in nutrient digestion and absorption, can be assessed using body indices, such as the Hepatopancreas Somatic Index (HPSI). The present study highlights that HSPI of shrimp was greatly influenced by water quality of the system. The increase in HPSI of shrimp might be attributed to the fact that biochar may improve in ionic balance of the rearing water (rise in alkalinity, potassium and magnesium level) causing more mineral deposition in the hepatopancreas to gradually increase its weight. The survival percentage of shrimps also showed a similar tendency, where the biochar treatments demonstrated high survival percentage compared to the control. This was in line with the findings reported by Jahan et al.^43^; *L. vannamei* reared in ISGW by supplementing the K^+^ and Mg^2+^concentrations in the feed and water had shown a higher survival rate as well as growth due to ion fortification as compared to diet.

Understanding shrimp’s digestive physiology and nutritional needs are made easier by measuring the enzyme activity in the digestive tract. Digestive enzymes in crustaceans are involved in the physiology of feeding, growth control^44^. In the digestive tract of shrimp, the production of the enzyme’s protease, amylase, and lipase is linked with the availability of the associated substrate^45^. The findings from the present study indicate that the activity of the enzyme amylase, protease, and lipase was significantly (p < 0.05) higher in the biochar treatments as compared to control. It may be observed that better ionic balance of system and optimum water quality trigger the higher metabolic activity pertaining to protein and carbohydrate digestion. Similarly, an elevated protease and amylase activity in *Penaeus vannamei* raised in inland saline ground water with sugarcane bagasse biochar-amended sediment is already documented by Jateen et al.^46^.

Aspartate transaminase (AST) alanine aminotransferase and (ALT) are enzymes that help metabolize amino acids. Improvements in appetite and feed consumption were facilitated by enhanced hepatopancreas health and increased production of AST and ALT enzymes^47^. AST catalyzes the interconversion of aspartate and α-Ketoglutaric to oxaloacetate and glutamate. The amino group from alanine is transferred by ALT to α-Ketoglutaric, which is then converted to glutamic acid to generate pyruvate. As a result, pyruvate and oxaloacetate are produced, which aid in the production of non-essential amino acids and protein synthesis and stimulate shrimp growth^47^. In the present study, the AST and ALT activity was studied in the hepatopancreas; the result showed that the mean AST and ALT value in biochar amended treatments showed higher significance than control. The highest value was recorded in T3, which is a mixed biochar treatment. Similar observations were made by Jateen et al. ^46^;an increase in AST activity in shrimp with the biochar-amended treatment groups.

The activities of SOD and CAT were also assessed in shrimp serum in the current study. The substances serve as the initial line of defense against dangers from the outside, particularly free radicals^48–50^. T-SOD inhibits lipid peroxidation by accelerating the disproportionation of the lipid peroxidation initiator^51^. Catalase is described as being able to decompose hydrogen peroxide and protect organisms against oxidative stress^52^. The dynamic equilibrium of free radical formation and elimination in organisms, according to Atli et al.^53^, avoids ROS-mediated tissue damage and membrane lipid peroxidation. In this study, CAT values are lower in biochar amended treatments, compared to control. SOD and CAT in biochar-amended treatments did not increase for 60 days experimental period as compared to control, indicating that biochar is essential in lowering the stress levels of organisms. This may be due improvement in soil and water quality of the system. In addition, it may also imply there is no negative impact of biochar on organisms in an aquaculture system^54^.

Of the total protein in crustacean hemolymph, hemocyanin, a copper-containing protein, makes up 60–95%^55^. It plays a role in numerous physiological processes, including the molt cycle, exoskeleton formation, osmoregulation, and protein storage. In this study, hemocyanin concentrations varied significantly in shrimp serum samples of all groups. A higher amount of ammonia reduced the OxyHc level which could be the reason a lower OxyHc concentration observed in control^56^. The enhanced hemocyanin concentration observed in all the biochar treatments indicate that farmed shrimps have sublime health and physiological status. The glucose level showed a significant decrease in treatments with biochar amendment, compared to the control.The decreased concentration may be due to the effective metabolic utilization of glucose by the cultured shrimp^18,57^^.^ However, the glucose level in the control group is elevated due to the stress-related increase in energy requirements^58^.

## Conclusion

The amendment of mixed biochar (PSB + BPB) into the sediment enhanced the water quality (reduction in ammonia-N, optimum calcium magnesium ratio) and it significantly improved the level of K^+^ which is a shortfall nutrient in inland saline water. Moreover, it increased the hemocyanin concentration, AST & ALT, protease, and amylase activity, which enhanced the growth and protein utilization of the cultured shrimp by reducing the FCR (within 1–1.26). In addition, SOD and CAT, which are oxidative stress enzymes, have shown lower values. The present study concludes that biochar technology can be a solution to improve the overall growth and health of *P. vannamei* culture systems in inland saline environments in addition to maintaining the quality of water which is a good example of environmentally friendly`aquaculture practice. The findings of this experiment are significant because low-quality water can be used for shrimp culture in the Inland saline system with amendment by biochar, which could be valuable for researchers addressing similar environmental challenges globally.

## Data Availability

All data underlying the results are available as part of the article. Raw data that supports the findings of this study are available from the corresponding author, upon reasonable request.

## Abbreviations

FRP: Fibre Reinforced Plastic
PSB: Paddy Straw Biochar
BPB: Banana Peduncle Biochar
PER: Protein Efficiency Ratio
FCR: Feed Conversion Ratio
HPSI: Hepatopancreatic Somatic Index
SGR: Specific Growth Rate
CAT: Catalase
SOD: Superoxide Dismutase
Ca: Calcium
Mg: Magnesium
K^+^: Potassium
FTIR: Fourier Transform Infrared (FTIR) Spectroscopy
IGSW: Inland Ground Saline Water
EDS: Energy-Dispersive X-ray Spectroscopy

## Declarations

### Permissions

Permissions were taken, while collecting the raw paddy straw from the farmers and banana peduncle waste from shopkeepers for conducting this study.
